# Beyond IHC Stain: Mitochondria as a Histochemical Biomarker for Apoptosis Detection in Hepatic Tissue Sections

**DOI:** 10.1111/ahe.70094

**Published:** 2026-02-16

**Authors:** Maria‐Cătălina Matei‐Lațiu, Andreea Muntean, Vasile Rus, Adrian Florin Gal

**Affiliations:** ^1^ Department of Histology, Faculty of Veterinary Medicine University of Agricultural Sciences and Veterinary Medicine Cluj‐Napoca Cluj‐Napoca Romania; ^2^ Department of Pharmacology, Faculty of Veterinary Medicine University of Agricultural Sciences and Veterinary Medicine Cluj‐Napoca Cluj‐Napoca Romania

**Keywords:** apoptosis, Heidenhain's iron haematoxylin, hepatocyte, histological staining, immunohistochemistry, mitochondria

## Abstract

Apoptosis plays a critical role in maintaining tissue homeostasis and preventing pathological conditions. The accurate detection of apoptosis is crucial for both research and diagnostic pathology, yet conventional histological staining methods often lack the sensitivity to identify early apoptotic changes. This study aimed to evaluate and compare the effectiveness of classical and emerging mitochondria‐targeting histological techniques in detecting apoptotic hepatocytes. Paraffinated hepatic tissue from five rabbits was analysed using haematoxylin–eosin (H&E), Goldner's trichrome (GT), immunohistochemistry for caspase‐3 (IHC‐Casp3) and Heidenhain's iron haematoxylin (HIH) staining. The percentage of apoptotic hepatocytes was quantified using two assessment methods, and statistical analyses determined the sensitivity of staining protocols. Histologically, the main apoptotic features identified in HIH‐stained hepatic specimens were fading ‘ghost’ mitochondria throughout the cytoplasm, chromatin condensation, fragmentation and dissolution and nucleolar margination. A similar proportion of apoptotic hepatocytes on the HIH staining compared to IHC‐Casp3 (19.00% ± 0.7 for HIH vs. 18.50% ± 0.7 for IHC‐Casp3) was observed. In comparison, significantly lower values were obtained in H&E (5.75%) and GT (7.5%) stains. The lack of statistical significance of HIH vs. IHC‐Casp3 demonstrates similar sensitivities. The additional quantitative analysis methods confirmed IHC as the most sensitive method (3.36 ± 3.82 apoptotic cells/field), followed by HIH, H&E, and GT. In situ histological evaluation of apoptosis remains challenging in standard H&E and GT‐stained sections. HIH stain, as a cost‐effective alternative to IHC, highlights early stages of apoptotic cells, providing a significant advantage over classical staining methods, emphasising their importance in diagnostic histopathology.

## 1 Introduction

Cell death is an irreversible biological process whereby the cell stops performing its vital functions. Up to the moment, three primary types of cell death have been described: apoptosis, necrosis and autophagy (Kroemer et al. 2009; D'Arcy 2019).

Apoptosis is a programmed and physiological form of cell death characterised by distinct morphological changes and regulated by various biochemical and enzymatic processes. The purpose of the process is to ensure the controlled elimination of cells without damaging surrounding structures. Furthermore, apoptosis is considered an active form of cell death, as it requires intrinsic signalling pathways to initiate self‐destruction in response to specific stimuli (D'Arcy 2019). On the other hand, necrosis is an uncontrolled, accidental, and irreversible yet now recognised regulated form of cell death triggered by external stressors, distinguished from apoptosis by its release of cellular debris and strong induction of inflammation (D'Arcy 2019; Edinger and Thompson 2004).

Biochemically, apoptosis involves several key changes, including protein cleavage, DNA fragmentation, and apoptotic body recognition by macrophages (Hengartner 2000). Protein and DNA fragmentation occur through the activation of caspases—cytosolic proteases that exist in an inactive form within normal cells. Once activated, caspases cleave vital cellular proteins and irreversibly dismantle organelles. Apoptosis becomes irreversible following caspase activation (Hu et al. 1998; Nakagawa et al. 2000; Koenig et al. 2001; Kang et al. 2002). In cases of irreversible cellular damage, viral infections or excessive oxidative stress, the tumour suppressor protein p53 halts the cell cycle and initiates apoptosis, thereby conserving essential nutrients for healthy cells and preventing the spread of viral infections. In adult animal organisms, the total number of cells remains relatively constant due to the equilibrium between cell proliferation and apoptosis, ensuring tissue homeostasis. When this balance is disrupted, excessive cell loss due to increased apoptosis or uncontrolled cellular proliferation may occur (Chandar and Viselli 2018).

Apoptosis involves energy‐dependent molecular cascades and occurs through three pathways: the extrinsic (death‐receptor) pathway, the intrinsic (mitochondrial cytochrome‐c‐regulated) pathway, and the perforin‐granzyme pathway activated by cytotoxic T cells. All pathways rely on caspase activation, and research indicates they are interconnected rather than operating independently (Elmore 2007).

In view of this, mitochondria play a crucial role in the process, being central regulators of apoptosis. This organelle orchestrates both intrinsic and extrinsic pathways of cell death. The intrinsic or mitochondrial, pathway is primarily governed by the balance between pro‐apoptotic and anti‐apoptotic proteins of the Bcl‐2 family, which control mitochondrial outer membrane permeabilisation (MOMP) and the release of apoptogenic factors such as cytochrome c, Smac/DIABLO, and apoptosis‐inducing factor (AIF) (Elmore 2007; Kierszenbaum and Tres 2020). The release of cytochrome c into the cytoplasm facilitates the formation of the apoptosome complex by binding to apoptotic protease activating factor‐1 (Apaf‐1) and procaspase‐9, leading to caspase activation and cell death (Martinvalet et al. 2005). On the other hand, the extrinsic apoptotic pathway is initiated by death receptors such as TNFR1 and Fas, which activate caspase‐8 and subsequently trigger the mitochondrial amplification loop via the pro‐apoptotic protein Bid (Miller and Zachary 2017).

Given the pivotal role of mitochondria in apoptosis, substantial research has focused on the identification of apoptotic markers and the development of histological techniques to detect mitochondrial‐mediated cell death. Traditional staining methods, including haematoxylin‐based approaches, provide valuable insights into apoptotic morphology, while immunohistochemical detection of cleaved caspases, cytochrome *c*, and mitochondrial structural alterations offers a more specific understanding of apoptosis at the molecular level (Hasui et al. 2012; Nambiar and Hegde 2016). More recently, alternative histochemical advanced assays were proposed and have provided novel perspectives on mitochondrial dysfunction during apoptosis (Gal et al. 2020).

In this study, we aim to explore and compare classical and emerging histological techniques for detecting apoptosis, with a focus on mitochondrial‐mediated cell death in hepatocytes. By evaluating the advantages and limitations of each method, we provide insights into their applicability in mitochondrial research and liver pathology.

## 2 Material and Methods

### 2.1 Biological Material

During the necropsy survey, hepatic tissue samples were harvested from five rabbits presented in the Pathology Department of the Faculty of Veterinary Medicine Cluj‐Napoca (Romania). The samples were collected only from individuals without any grossly visible hepatic lesions, being selected only the rabbits in which the hepatic function was not affected (all individuals died due to severe respiratory distress).

### 2.2 Histological and Immunohistochemical Assessment

#### 2.2.1 Samples Preparation

Each hepatic tissue sample was divided into two halves and fixed with either 10% buffered formalin or Kolster's fixative. Samples were routinely processed for paraffin embedding and sectioned at 3–5‐μm thickness using a rotary microtome (Leica RM2125, Germany). The mounted samples were stained with four different staining protocols, as follows: the samples fixed with 10% buffered formalin were stained with two routine staining methods (haematoxylin–eosin and Goldner's trichrome) and an immunohistochemical reaction (anti‐caspase 3 antibody). In contrast, the samples fixed with Kolster's mixture were stained with Haidenheim's iron haematoxylin staining (a histochemical reaction that targets and stains the mitochondria).

#### 2.2.2 Samples Staining

For the haematoxylin–eosin (H&E) and Goldner's trichrome (GT) stainings, the 5 μm sections were dewaxed and hydrated and after subjected to the standard staining protocols (Suvarna et al. 2019).

Immunohistochemistry was performed to detect caspase‐3 expression using specific antibodies (Cleaved Caspase‐3 (Asp 175) (5A1E), CellSignal). Three‐micron sections were mounted on poly‐l‐lysine‐coated slides and then maintained for 1 h at 60°C and 24 h at 37°C. The next step was to dewax the samples in xylene and hydrate them with alcohol series baths. The antigen retrieval of epitopes was performed by the heat method in a sodium citrate bath, pH 6.0 (Taylor et al. 1996). The endogenous peroxidase blockage was performed with a 3% sodium peroxide solution (Elabscience 2‐step plus Poly‐HRP Anti Rabbit/Mouse IgG Detection System with DAB Solution). Afterwards, the incubation of the monoclonal antibodies for 20 h at 4°C in a humid chamber was performed. The 1:2000 antibody dilution was used, according to the manufacturer's instructions. Subsequently, the detection kit provided by Elabscience (2‐step Plus Poly‐HRP Anti Rabbit/Mouse IgG Detection System) was applied to develop the IHC (immunohistochemical) reaction. The tissue samples were then exposed to diaminobenzidine (DAB), and, later, the counterstaining was performed with Mayer's haematoxylin (Suvarna et al. 2019).

To visualise the cytosolic mitochondria, the harvested hepatic tissue specimens underwent a special fixation followed by a distinctive histochemical stain with Heidenhain's iron haematoxylin. Since the fixation represents a decisive step in the proper preservation of mitochondria, the tissue samples were subjected to Kolster's fixative (Gal et al. 2020; Gabe 1968; Locquin and Langeron 1983). Heidenhain's iron haematoxylin solution was prepared by using 10% haematoxylin in 96% alcohol and ripened for 4 weeks before use. The 4 μm samples displayed on slides were dewaxed and hydrated with seriated alcohol baths, treated with iron alum for 6 h, washed with distilled water, and immersed in stained iron haematoxylin solution up to the next day. The staining regression was realised with iron alum under microscopical control, which was later followed by counterstaining with eosin for 20 s (Suvarna et al. 2019).

#### 2.2.3 Samples Examination

All the obtained coloured slides were assessed with the aid of an optical microscope (Olympus BX‐41, Olympus, Japan), and the microphotographs were achieved with the aid of a digital camera (Olympus SC180, Olympus, Japan) and CellSens Entry 3.1 (Olympus, Japan).

#### 2.2.4 Apoptotic Scores

The percentage (%) of apoptotic hepatocytes (AH) was assessed by counting the hepatocytes that displayed evident apoptotic features out of a total of 200 hepatic cells and applying the following formula: AH% = (100*AH)/200. However, the second counting method mattered in the microscopic examination of 50 consecutive high‐power fields (HPF) (400× magnification degree), in which the AHs were counted for each field. The two scoring methods were applied on all the obtained samples for all four staining procedures used, and the assessment was done independently by two experienced histologists.

#### 2.2.5 Statistical Analysis

Values obtained for each staining were compared to assess statistically significant differences. Percentages were analysed using ordinary one‐way ANOVA followed by Tukey's multiple comparisons test (*p* < 0.05). Values from the second counting method were evaluated with descriptive statistics and tested for normality using the Shapiro–Wilk test. Data with abnormal distribution were compared using a Kruskal–Wallis test (*p* < 0.05). Dunn's multiple comparisons test was used to determine the accuracy ratio for apoptosis detection across all histological, histochemical, and immunohistochemical procedures. All statistical analyses were performed in Microsoft Excel 2016 and GraphPad Prism 8.0.1.

## 3 Results

**FIGURE 1:**
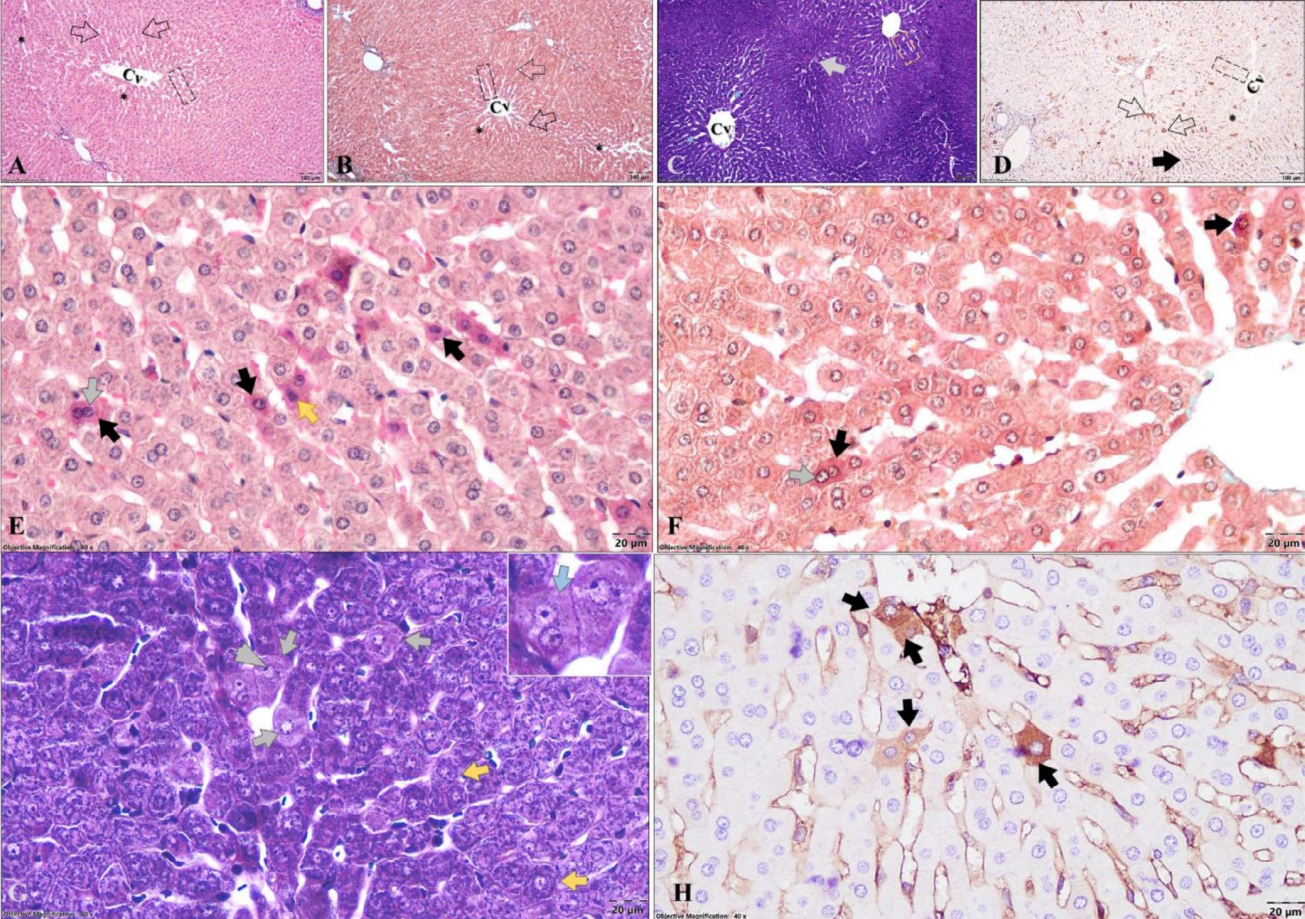
Apoptosis detection in hepatic parenchyma by diverse staining methods. (A, B) Overview of the hepatic microanatomy (40× magnification) stained with two routine methods (A—H&E; B—GT) illustrating hepatocytes (empty arrows) arranged as Remak chords (dashed rectangle) oriented towards the centrilobular venule (CV), and sinusoid capillaries interposed between Remak chords (*); (C) HIH stain of the hepatic parenchyma displaying the common features (e.g., Remak chords—dashed rectangle, the thin walled sinusoid capillaries—blue asterisck (*), and centrilobular vein—Cv), and isolated prominent apoptotic hepatocytes (grey arrow) exhibiting a lighter homogenous cytoplasm showing fading ‘ghost’ mitochondria feature comparing to interphase hepatocytes that display prominent granular to rod‐like mitochondria throughout the cytoplasm (40× magnification); (D) IHC‐Casp 3 reaction of the hepatic parenchyma displaying immunolabelled apoptotic hepatocytes (empty arrows) scattered between hepatocytes during interphase (black arrow), which are arranged in cords of Remak (dashed rectangle), separated by sinusoid capillaries (*) that converge towards the centrilobular venule (Cv) (40× magnification); (E, F) Microscopic features of apoptotic hepatocytes in regular H&E and GT stains, showing intense acidophilic staining of the cytoplasm (black arrows), and nuclear cortical hyperchromatosis (yellow arrow; 400× magnification); (G) HIH stained hepatic parenchyma displaying a marked difference between apoptotic hepatocytes (grey arrows), which present an homogenous cytoplasm due to fading ‘ghost’ mitochondria feature (blue arrow), and functional hepatocytes (yellow arrows) that include prominent granular to rod‐like mitochondria throughout the cytoplasm (400× magnification); nuclear changes specific to apoptosis, such as nucleolar margination (arrowhead), can be observed in HIH staining; (H) Immunohistochemical features of IHC‐Casp3 reaction presenting immunolabelled apoptotic hepatocytes with a conspicuous dark‐brown cytoplasm (black arrows; 400× magnification).

The hepatic parenchyma showed normal histoarchitecture in all specimens examined. Accordingly, polygonal mononucleated/binucleated hepatocytes displayed as tortuous (Remak) chords radiating out of the centrilobular vein were noticed. In between the Remak chords, the sinusoid capillaries merge in the centrilobular vein; next to the hepatic lobules, normal portal canals were observed. Despite the staining method used, all the aforementioned details of the hepatic lobules were evident (Figure 1A–D).

On the two routine staining methods (H&E and GT), in the hepatic parenchyma scattered AH were present. The cells that underwent cell death exhibited characteristic morphological features in both the cytoplasm and the nucleus. Accordingly, at the cytoplasmic level a condensation of the material was observed, microscopically represented by an intense acidophilic staining. The nuclear changes were represented by cortical hyperchromatosis (in an earlier stage of apoptosis), followed by pyknotic nuclei, or even karyorrhexis or karyolysis (Figure 1E,F). However, the aforementioned description is representative of the late stages of apoptosis.

Using HIH staining, as compared to living hepatocytes that displayed prominent granular to rod‐like mitochondria throughout the cytoplasm (Gal et al. 2020; Gabe 1968; Locquin and Langeron 1983; Suvarna et al. 2019), the scattered AH exhibited a lighter, more homogenous cytoplasm with fading ‘ghost’ mitochondria. Additionally, discrete halos corresponding to fading mitochondria were also observed within the cytoplasm of AH (Figure 1G). The preceding histochemical features are attributable to mitochondrial degradation and loss. Interestingly, as compared to regular H&E and GT stains, minimal nuclear changes were identified in the hepatocytes with ‘ghost’ mitochondria features. The nuclear changes were represented by chromatin condensation and fragmentation along with its margination, followed by dissolution. The previous nuclear changes were often associated with nucleolar margination. The discrete nuclear changes may suggest early stages of apoptosis. Most likely, the altered appearance of apoptotic cells is influenced by biochemical changes that impair the mitochondria's ability to retain ferric haematoxylin.

Under the immunohistochemical reaction, the cytoplasm of AH exhibited brown staining, indicating the specific binding of anti‐caspase‐3 antibodies to the caspase‐3 enzyme. The brown cytoplasmic staining in apoptotic cells resulted from peroxidase‐catalysed reaction with diaminobenzidine (DAB; Figure 1H). In many immunolabelled AH, negligible nuclear changes were identified, suggesting high sensitivity of the IHC reaction, including for the early stages of apoptosis.

**FIGURE 2:**
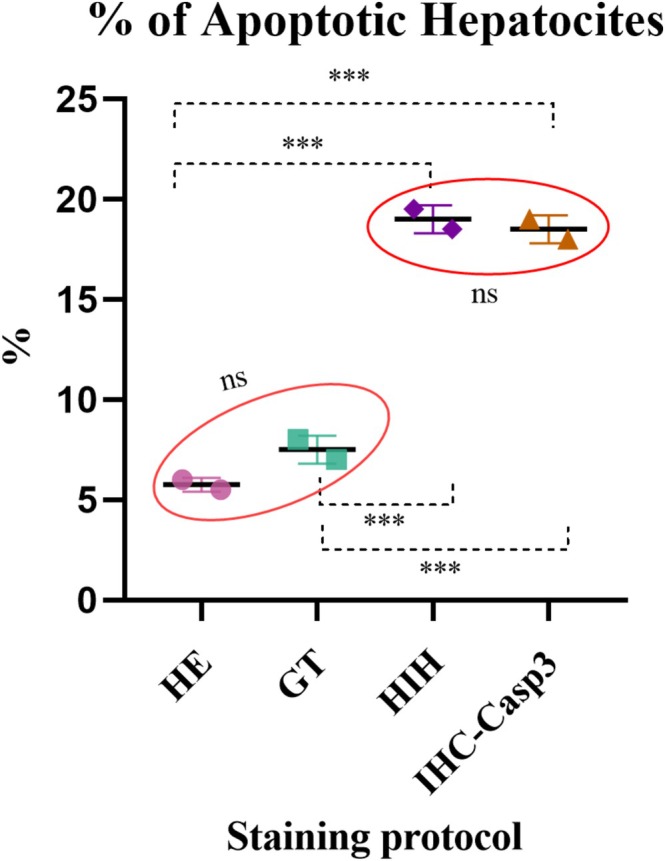
Comparative assessment of the AH percentage in the four staining protocols (expressed as the two values obtained by the two evaluators); ***p ≤ 0.001 according to Tukey's multiple comparisons test; ns—without statistical significance according to Tukey's multiple comparisons test.

Significant differences were observed in the percentage of AH identified by the four staining methods. Accordingly, the IHC‐Casp3 and HIH staining protocols demonstrated a higher sensitivity to label apoptotic cells, resulting in a similar apoptotic percentage (i.e., 19% for HIH and 18.5% for IHC‐Casp3). The two standard histological staining procedures marked a significantly lower percentage of AH compared to IHC‐Casp3/HIH protocols (i.e., 5.75% for H&E and 7.5% for GT; Figure 2; Table 1).

**TABLE 1:** AH scoring methods presented as mean ± standard deviation of mean.

	Staining protocols			
	H&E	GT	HIH	IHC‐Casp3
Score 1 (AH%)	5.75 ± 0.35	7.50 ± 0.7	19.00 ± 0.7	18.50 ± 0.7
Score 2 (AH/field)	1.28 ± 1.97	0.85 ± 1.25	1.75 ± 1.8	3.36 ± 3.82

Abbreviations: AH, apoptotic hepatocytes; GT, Goldner's trichrome stain; HE, haematoxylin–eosin stain; HIH, Heidenheim iron haematoxylin; IHC‐Casp3, Immunohistochemical reaction for anti‐caspase 3 antibody.

**FIGURE 3:**
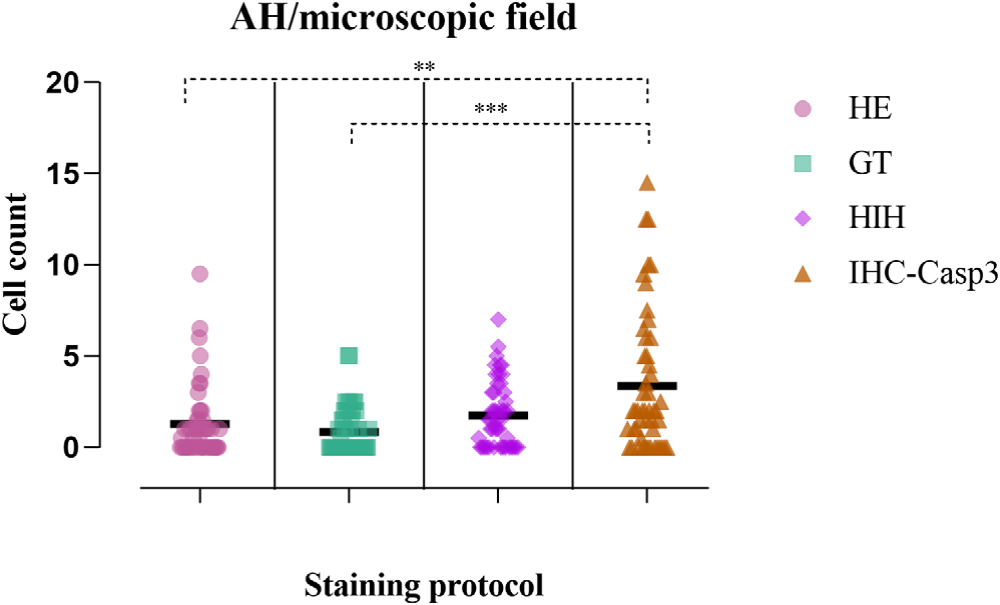
Counted apoptotic hepatocytes (AH) in 50 consecutive HPF assessed on the four staining methods, presented as individual values; **p ≤ 0.01 according to Dunn's multiple comparisons test; ***p ≤ 0.001 according to Dunn's multiple comparisons test.

However, the second quantitative assessment of AH revealed that the immunohistochemical protocol demonstrated the highest sensitivity (3.36 ± 3.82), while the lowest was manifested by GT routine staining (0.85 ± 1.25 cells/field). Moreover, according to Dunn's multiple comparisons test, neither the differences recorded between the routine staining methods nor the ones with the histochemical protocol were statistically significant (Figure 3; Table 1).

## 4 Discussion

In recent decades, apoptosis detection has extended beyond traditional optical and electron microscopy methods. DNA fragmentation resulting from apoptotic cell death can be detected through agarose gel electrophoresis, while flow cytometry has been employed to distinguish apoptotic from necrotic cells (Bardales et al. 1997; Dolzhanskiy and Basch 1995). Additional methodologies, though costly, include fluorescence‐activated cell sorting (FACS), immunoblotting, high‐performance liquid chromatography (HPLC), TUNEL (Terminal deoxynucleotidyl transferase dUTP Nick‐End Labelling) assay, and immunofluorescence for detecting mitochondrial intermembrane space proteins (Bardales et al. 1997). Although highly specific and sensitive, these methods are limited in assessing apoptosis in tissue sections because they do not provide a comprehensive microscopic overview of apoptotic cell distribution (Galluzzi et al. 2007). Given this, the assessment of apoptosis on tissue slides has been extensively studied through various methodologies, each presenting distinct advantages and limitations. Traditional histological techniques, such as H&E and GT methods, have been widely used because they are considered fast, inexpensive, and fairly reliable for detecting apoptotic cells. However, this is not entirely true since their sensitivity in detecting apoptotic changes remains suboptimal (Gal et al. 2020), underestimating the apoptosis ratio by two to three folds (Garrity et al. 2003; Galluzzi et al. 2007). In contrast, immunohistochemistry (IHC), a standard tool in pathological anatomy (Lin and Chen 2014), provides superior visualisation of apoptotic features by marking early stages of apoptosis. Still, it must not be forgotten that microanatomy provided by routine staining methods represents the gold standard for the endorsement of new techniques (Garrity et al. 2003).

In an attempt to promote a rapid and fairly low‐cost technique for the detection and quantification of apoptotic cells on histological tissue sections (Gal et al. 2020), we refered to the accuracy of the HIH histochemical technique on hepatic tissue. The results revealed a similar proportion of apoptotic hepatocytes on the HIH staining compared to IHC‐Casp3 (19.00% ± 0.7 for HIH vs. 18.50% ± 0.7 for IHC‐Casp3). The lack of statistical significance of HIH vs IHC‐Casp3 proves that both methods offer a similar sensitivity (*p* > 0.05). Across the four methods used to identify apoptotic figures in the hepatic parenchyma, IHC‐Casp 3 and HIH showed a clear advantage over H&E and GT. This finding is consistent with previous reports indicating that classical stains cannot reliably distinguish apoptotic cells, especially in early stages (Galluzzi et al. 2007). Based on the quantitative analyses, the effectiveness of the staining techniques ranks as follows: IHC, HIH, H&E and TG. Although IHC and HIH display comparable specificity, the choice between them remains context‐dependent. Economic considerations may favour HIH in certain situations, despite its slightly lower sensitivity.

Histological evaluation of apoptosis remains challenging, particularly in its early stages, where morphological changes are subtle and difficult to identify in H&E and GT‐stained sections. As a backup to immunohistochemistry, HIH staining proves an economical and yet valuable technique by enabling the histochemical visualisation of mitochondrial state (Gal et al. 2020; Gabe 1968; Locquin and Langeron 1983; Suvarna et al. 2019), indirectly facilitating the spotting of apoptotic hepatocytes.

The primary microscopic features observed in HIH‐stained apoptotic hepatocytes include a brighter and homogenous cytoplasm due to the fading ‘ghost’ mitochondria feature. Interestingly, minimal nuclear changes were identified in most of the hepatocytes with ‘ghost’ mitochondria features due to their degradation and loss in the early phases of apoptosis. As the apoptotic process progresses, HIH‐stained tissue specimens also displayed nuclear alterations such as chromatin condensation, fragmentation and margination, along with nucleolar margination. In mid to late apoptosis, regular microscopic features detectable as well on H&E/GT stains may occur, namely karyopyknosis, karyolysis and karyorrhexis. These align with prior studies indicating that chromatin margination and fragmentation, cellular contraction, and apoptotic body formation characterise apoptosis (Zeiss 2003).

Mitochondria play pivotal roles in key cellular activities, starting with cellular metabolism, energy production and cellular signalling (Martínez‐Reyes and Chandel 2020). However, mitochondrial dysfunction contributes to aging and a range of age‐related diseases (e.g., neurodegenerative, cardiovascular, and metabolic disorders), whereas its role in the regulation of cell fate by mediation of mitochondria‐associated programmed cell death is undeniable. The fundamental role of mitochondria in activating apoptosis and other forms of mitochondria‐associated programmed cell death, including necroptosis, pyroptosis, ferroptosis, parthanatos, and paraptosis is well‐known in mammals (Kroemer et al. 2009; Wang and Youle 2009; Nguyen et al. 2023). Consequently, due to their irreplaceable roles, mitochondria have captured the attention in recent decades, fact evidenced by more than 10,000 research papers published annually (Oh et al. 2020; Nguyen et al. 2023).

## 5 Conclusion

In conclusion, our paper shows mitochondria as a valuable tool for apoptotic figure detection in histological tissue specimens through their histochemical marking using the HIH stain. HIH stain, as a cost‐effective alternative to IHC, highlights earlier stages of apoptotic cells compared with routine staining, providing a significant advantage over them, emphasising their importance in diagnostic histopathology. Still, future similar reports should aim to refine these methodologies further, potentially integrating molecular techniques to enhance apoptotic cell identification and characterisation.

## Author Contributions

Maria‐Cătălina Matei‐Lațiu: writing – review and editing, writing – original draft, visualisation, resources, investigation, formal analysis, conceptualisation, project administration. Andreea Muntean: writing – review and editing, investigation. Vasile Rus: writing – review and editing, visualisation, resources, investigation, conceptualisation. Adrian Florin Gal: writing – review and editing, visualisation, supervision, resources, project administration, investigation, conceptualisation.

## Conflicts of Interest

The authors declare no conflicts of interest.

## Data Availability

The data that support the findings of this study are available from the corresponding author upon reasonable request.
